# Fucoxanthin Enhances Cisplatin-Induced Cytotoxicity via NFκB-Mediated Pathway and Downregulates DNA Repair Gene Expression in Human Hepatoma HepG2 Cells

**DOI:** 10.3390/md11010050

**Published:** 2013-01-08

**Authors:** Cheng-Ling Liu, Yun-Ping Lim, Miao-Lin Hu

**Affiliations:** 1 Department of Food Science and Biotechnology, National Chung Hsing University, Taichung 402, Taiwan; E-Mail: liouoiu@msn.com; 2 Department of Pharmacy, College of Pharmacy, China Medical University, Taichung 404, Taiwan; E-Mail: limyp@mail.cmu.edu.tw; 3 Department of Emergency, Toxicology Center, China Medical University Hospital, Taichung 404, Taiwan; 4 Agricultural Biotechnology Center, National Chung Hsing University, Taichung 402, Taiwan

**Keywords:** fucoxanthin, cisplatin, NFκB, DNA repair, MAPK, PI3K/AKT

## Abstract

Cisplain, a platinum-containing anticancer drug, has been shown to enhance DNA repair and to inhibit cell apoptosis, leading to drug resistance. Thus, the combination of anticancer drugs with nutritional factors is a potential strategy for improving the efficacy of cisplatin chemotherapy. In this study, we investigated the anti-proliferative effects of a combination of fucoxanthin, the major non-provitamin A carotenoid found in *Undaria Pinnatifida*, and cisplatin in human hepatoma HepG2 cells. We found that fucoxanthin (1–10 μΜ) pretreatment for 24 h followed by cisplatin (10 μΜ) for 24 h significantly decreased cell proliferation, as compared with cisplatin treatment alone. Mechanistically, we showed that fucoxanthin attenuated cisplatin-induced NFκB expression and enhanced the NFκB-regulated Bax/Bcl-2 mRNA ratio. Cisplatin alone induced mRNA expression of excision repair cross complementation 1 (ERCC1) and thymidine phosphorylase (TP) through phosphorylation of ERK, p38 and PI3K/AKT pathways. However, fucoxanthin pretreatment significantly attenuated cisplatin-induced ERCC1 and TP mRNA expression, leading to improvement of chemotherapeutic efficacy of cisplatin. The results suggest that a combined treatment with fucoxanthin and cisplatin could lead to a potentially important new therapeutic strategy against human hepatoma cells.

## 1. Introduction

Hepatocellular carcinoma (HCC) is the major cause of cancer death in Taiwan and one of the most common cancers in the world, accounting for approximately 6% of all human cancers [1,2]. For most patients with unresectable HCC, chemotherapy plays an important role in the treatment of HCC [3]. Unfortunately, chemotherapy has limited effect on survival owing to multiple drug resistance (MDR) [4]. Much evidence indicates that the MDR is involved in drug efflux, DNA repair activity, and altered survival and apoptotic signaling pathways [5]. Thus, the new therapeutic strategies for malignant HCC using combined therapies or combined agents with distinct molecular mechanisms are considered more promising for higher therapy efficacy, resulting in better survival.

Platinum antitumor compounds, such as cisplatin (*cis*-diamminedichloroplatinum (II)) and its analogs, have been used widely as a chemotherapeutic drug for a variety of malignancies including hepatocellular carcinoma [6]. Platinum can directly bind to intra- and inter-strand DNA molecules to form predominantly platinum-DNA adducts that ultimately interfere with DNA transcription and replication and result in cell death [7,8,9]. Although cisplatin is extensively used in chemotherapy, its effectiveness is limited by acquired or intrinsic resistance [10]. A number of mechanisms of cisplatin resistance in cancer cells has been recognized: (1) increased reflux; (2) increased inactivation by sulfhydryl molecules such as glutathione; (3) altered expression of proteins in signal transduction pathways that control apoptosis; and (4) increased DNA repair [11]. DNA repair pathways that may result in platinum-based chemotherapeutic resistance include mismatch repair (MMR) and nucleotide excision repair (NER) [12,13]. Excision repair cross-complementation group 1 (ERCC1) is the initial enzyme in the NER pathway of DNA repair, and reports have shown that increased mRNA levels of ERCC1 are associated with clinical resistance to platinum-based chemotherapy in human lung, gastric, ovarian, cervical, and colorectal carcinomas and impact with the survival rate of cancer patients [14,15,16,17,18]. In addition, thymidine phosphorylase (TP), a key enzyme in the pyrimidine nucleoside salvage pathway, is known to catalyze the reversible conversion of thymidine to thymine and 2-deoxy-D-ribose-1-phosphate [19]. TP expression in various kinds of tumors is higher than that in the adjacent non-neoplastic tissues [20], and TP-overexpressed cell lines are more resistant to various apoptosis-inducing stimuli such as cisplatin and microtubule-interfering agents, hypoxia, and Fas ligands [21,22,23].

Nuclear transcription factor kappa B (NFκB), a heterodimeric protein composed of different combinations of members of the Rel family of transcription factors, is inactivated in the cytoplasm by IκBs, a class of inhibitor proteins. Phosphorylation of IκB by upstream kinases promotes its ubiquitination-dependent degradation, allowing NFκB to translocate to the nucleus and induce target genes, which is associated with cell proliferation [24], angiogenesis [25], metastasis [26], suppression of apoptosis [27], promotion of oncogenesis [28], and cancer therapy resistance [24]. NFκB is known to inhibit apoptosis through induction of anti-apoptotic proteins or suppression of pro-apoptotic genes, and the ratio of pro- and antiapoptotic Bcl-2 family members is critical to determine cell susceptibility to apoptotic insults [29,30]. Much evidence indicates that several anticancer drugs such as cisplatin, docetaxel, gemcitabine induce NFκB nuclear translocation and activation of its target genes, thereby potentially leading to chemoresistance [31]. For example, it has been reported that increased resistance of human cervical carcinoma cells to cisplatin is partly mediated via enhancement of cisplatin-induced NFκB activation [32]. Therefore, agents capable of inhibiting NFκB function might be considered as an adjuvant approach in combination with chemotherapeutic agents for a variety of cancers. 

Fucoxanthin is one of the most abundant carotenoids and contributes more than 10% of the estimated total production of carotenoids in nature [33]. This carotenoid has been shown to have several biological functions, such as antioxidant activity [34,35,36,37], anti-obese effect [38,39], antidiabetic activity [40], antimutagenicity [41], anti-inflammation [42,43], and anticancer effects [44,45]. A previous report revealed that inhibition of proliferation of human hepatoma HepG2 cells by fucoxanthin is related to cell cycle arrest by downregulation of cyclin D and induction of GADD45A gene expression [45,46,47]. We recently reported that fucoxanthin exhibits anti-drug resistance potential and that the effect is likely associated with attenuated interaction between pregnane X receptor (PXR) and coactivator (SRC-1), thereby potentially preventing activation of PXR-mediated CYP3A4 and MDR1 expression [48]. As the combination of anticancer drugs with nutritional factors is a potential strategy for improving the efficacy of chemotherapy, we herein employed human hepatoma HepG2 cells to determine whether a combination of fucoxanthin and cisplatin may enhance the inhibition of cell proliferation.

## 2. Results

### 2.1. Fucoxanthin Increases the Sensitivity of Cisplatin in HepG2 Cells

**Figure 1:**
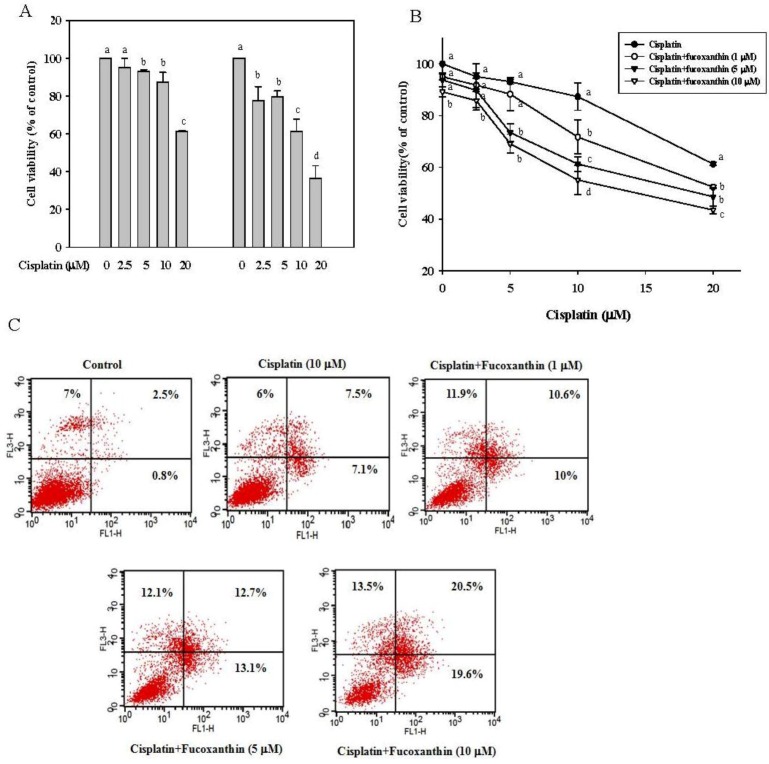
Effects of cisplatin (2.5–20 μM) alone or in combination with fucoxanthin (1–10 μM) on cell viability of human hepatoma HepG2 cells. (**A**) Cell viability of HepG2 cells incubated with cisplatin for 24 and 48 h. (**B**) Cell viability of HepG2 cells incubated with cisplatin (2.5–10 μM) for 24 h after pretreatment with fucoxanthin (1–10 μM) for 24 h. (**C**) Apoptotic cells in HepG2 cells incubated with cisplatin (2.5–10 μM) for 24 h after pretreatment with fucoxanthin (1–10 μM) for 24 h. Values are means ± SD, *n* = 3; means without a common letter differ significantly, *P* < 0.05.

InHepG2 cells treated with cisplatin (2.5–20 μM) for 24 and 48 h, (Figure 1A), we found that cisplatin significantly inhibited the cell viability (13% at 24 h and 39% at 48 h, *P* < 0.05, respectively, at 10 μM cisplatin). In addition, fucoxanthin significantly inhibited the cell proliferation of HepG2 by 17% and 28% after incubation with 10 μM fucoxanthin for 24 h and 48 h, respectively (Figure S1). To investigate whether fucoxanthin increases the sensitivity of cisplatin in HepG2 cells, we pre-incubated HepG2 cells with fucothanxin (1–10 μM) for 24 h followed by incubation with cisplatin (2.5–20 μM) for 24 h. Results reveal that the cell viability of HepG2 cells was significantly and concentration-dependently inhibited (Figure 1B), with an inhibition of 37% at 10 μM fucoxanthin and 10 μM cisplatin, as compared with cisplatin treatment alone. In addition, the combination of fucoxanthin with cisplatin increased early apoptotic cells (PI negative, Annexin V-FITC positive) and late apoptotic cells (PI positive, Annexin V-FITC positive) (Figure 1C). The results indicate that fucoxanthin enhances the anti-proliferative effect of cisplatin in human hepatoma HepG2 cells.

### 2.2. Fucoxanthin Attenuates the NFκB Expression Induced by Cisplatin and Restores the Phosphorylation of IκB-α Inhibited by Cisplatin

**Figure 2:**
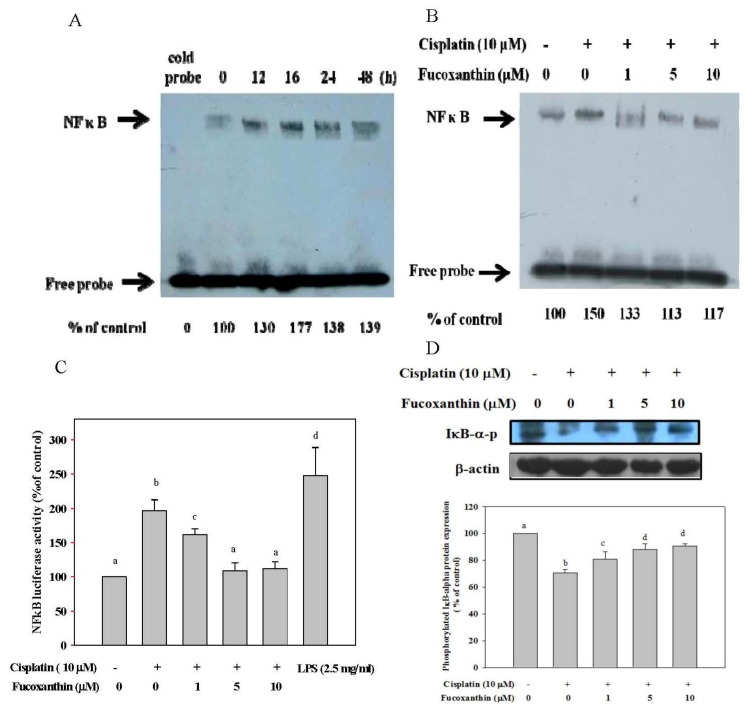
NFκB expression in HepG2 cells pretreated with fucoxanthin (0–10 μM) followed by incubation with cisplatin (10 μM). (**A**) NFκB expression in HepG2 cells incubated with cisplatin (10 μM) for 0–48 h; (**B**) NFκB expression in HepG2 cells incubated with cisplatin (10 μM) for 16 h after pretreatment with fucoxanthin (1–10 μM) for 24 h; (**C**) NFκB luciferase activity expression in HepG2 cells incubated with cisplatin (10 μM) for 16 h after pretreatment with fucoxanthin (1–10 μM) for 24 h; (**D**) Phosphorylation of IκBin HepG2 cells incubated with cisplatin (10 μM) for 16 h after pretreatment with fucoxanthin (1–10 μM) for 24 h. Values are means ± SD, *n* = 3; means without a common letter differ significantly, *P* < 0.05.

We also evaluated the effect of fucoxanthin on NFκB expression induced by cisplatin in HepG2 cells, as determined by the EMSA and NFκB reporter gene assays. As shown as in Figure 2A, cisplatin μM) most strongly induced NFκB binding activity at 16 h of incubation (by 77%, as compared with untreated cells). However, fucoxanthin concentration-dependently attenuated cisplatin-induced NFκB binding activity, with a 37% inhibition at 5 μM fucoxanthin (Figure 2B). We also showed that fucoxanthin significantly and concentration-dependently attenuated cisplatin-induced NFκB luciferase activity in a similar pattern to that of NFκB binding activity (Figure 2C). In addition, fucoxanthin significantly and concentration-dependently restored cisplatin-inhibited IκB-α***-***phosphorylation in HepG2 cells at 24 h of incubation, as compared with cisplatin treatment alone (Figure 2D). 

### 2.3. Fucoxanthin Combined with Cisplatin Increases the Ratio of Bax/Bcl-2 mRNA Expression in HepG2 Cells

**Figure 3:**
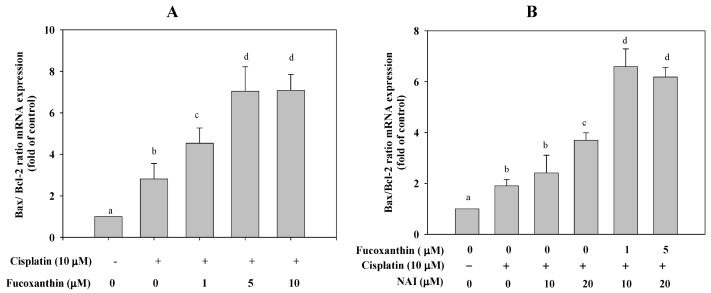
(**A**) The ratio of Bax/Bcl-2 mRNA in HepG2 cells pretreated with fucoxanthin (1–10 μM) for 24 h followed by incubation with cisplatin (10 μM) for 24 h. (**B**) The ratio of Bax/Bcl-2 mRNA in HepG2 cells pretreated with fucoxanthin (5 μM) for 24 h followed by incubation with NFκB activation inhibitor (NAI, 20 μΜ) for 2 h and then treated cisplatin (10 μM) for 24 h. Values are means ± SD, *n* = 3; means without a common letter differ significantly, *P* < 0.05.

Treatment of HepG2 cells with cisplatin (10 μM) for 24 h significantly increased the ratio of Bax/Bcl-2 mRNA expression (by 1.8-fold, *P* < 0.001, as compared with untreated cells). However, pretreatment of HepG2 cells with fucoxanthin for 24 h followed by incubation with cisplatin for 24 h significantly and concentration-dependently increased the ratio of Bax/Bcl-2 mRNA expression (by 4.3 fold, *P* < 0.001, as compared with cisplatin treatment alone) (Figure 3A). To further determine whether fucoxanthin in combination with cisplatin enhances the ratio of Bax/Bcl-2 mRNA primarily through NFκB-regulated pathways, we pretreated HepG2 cells with fucoxanthin for 24 h followed by incubation with an NFκB activation inhibitor (NAI) (10 and 20 μM) for 2 h and then by incubation with cisplatin (10 μM) for 24 h. We found that the combination of fucoxanthin, NAI and cisplatin synergistically or additively increased the ratio of Bax/Bcl-2 mRNA expression, as compared with the NFκB activation inhibitor alone (Figure 3B). Thus, the data indicate that fucoxanthin increases the ratio of Bax/Bcl-2 mRNA expression and that this effect is likely associated with inhibition of the NFκB pathway.

### 2.4. Fucoxanthin Attenuates mRNA Expression of ERCC1 and TP Induced by Cisplatin

**Figure 4:**
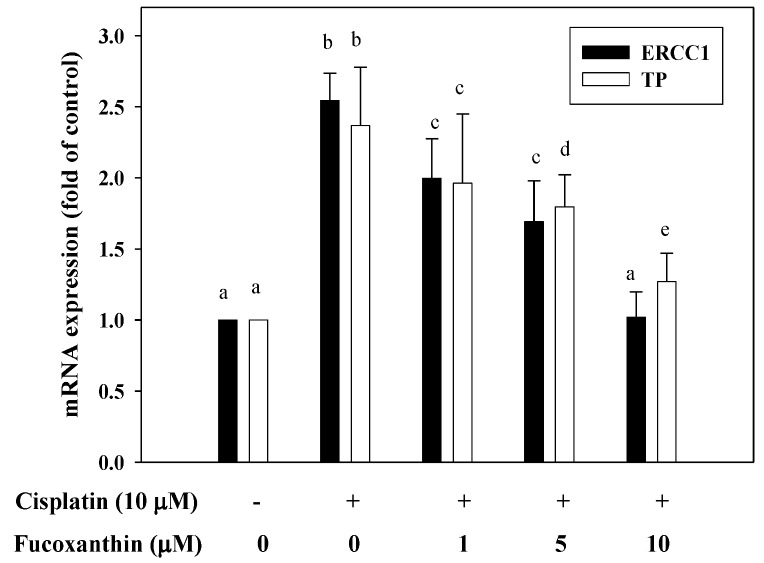
The level of ERCC1 and TP mRNA in HepG2 cells pretreated with fucoxanthin (1–10 μM) for 24 h followed by incubation with cisplatin (10 μM) for 24 h. Values are means ± SD, *n* = 3; means without a common letter differ significantly, *P* < 0.05.

Real-time PCR was performed to evaluate the mRNA levels of ERCC1 and TP. As shown in Figure 4, cisplatin (10 μM) treatment alone significantly increased the mRNA expression of ERCC1 and TP in HepG2 cells. However, the induced mRNA expression of ERCC1 and TP in HepG2 cells by cisplatin (10 μM) was attenuated by pretreatment with fucoxanthin (1–10 μM) for 24 h, with a 1.5-fold and a 1.2-fold inhibition, respectively, at 10 μM fucoxanthin, as compared with cisplatin treatment alone. 

### 2.5. Fucoxanthin Attenuates the Phosphorylation of ERK1/2, p38, AKT and PI3K in HepG2 Cells

**Figure 5:**
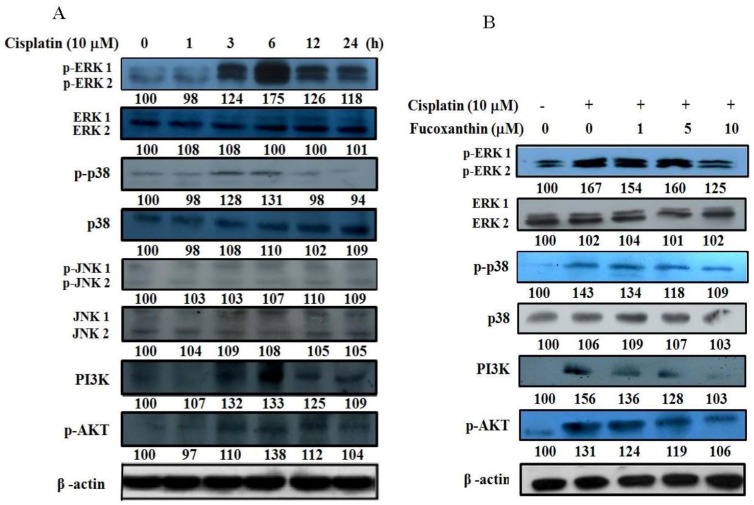
The protein expression of MAPK family and PI3K/AKT in HepG2 cells pretreated with fucoxanthin (0–10 μM) followed by incubation with cisplatin (10 μM). (**A**) The protein expression of MAPK family and PI3K/AKT in HepG2 cells incubated with cisplatin (10 μM) for 0–24 h. (**B**) The protein expression of MAPK family and PI3K/AKT in HepG2 cells incubated with cisplatin (10 μM) for 6 h after pretreatment with fucoxanthin (1–10μM) for 24 h.

The time effect of cisplatin on protein expression of the mitogen-activated protein kinase (MAPK) family (p38, ERK, and JNK) and phosphatidylinositol 3-kinase (PI3K)/AKT in HepG2 cells were determined by Western blotting. Results reveal that cisplatin (10 μM) markedly increased the phosphorylation of ERK, p38 and PI3K/AKT at 6 h of incubation, but it did not affect the phosphorylation of JNK or the protein expression of ERK, p38 and JNK (Figure 5A). We then determined whether pretreatment of HepG2 cells with fucoxanthin (1–10 μM) for 24 h attenuates the induction of MAPK family and PI3K/AKT protein expression by cisplatin (10 μM). We found that fucoxanthin concentration-dependently attenuated cisplatin-induced phosphorylation of ERK, p38 and PI3K/AKT (Figure 5B). 

### 2.6. Effect of Fucoxanthin in Combination with ERK, p38 and PI3K Inhibitor on ERCC1 and TP mRNA Expression in HepG2 Cells

**Figure 6:**
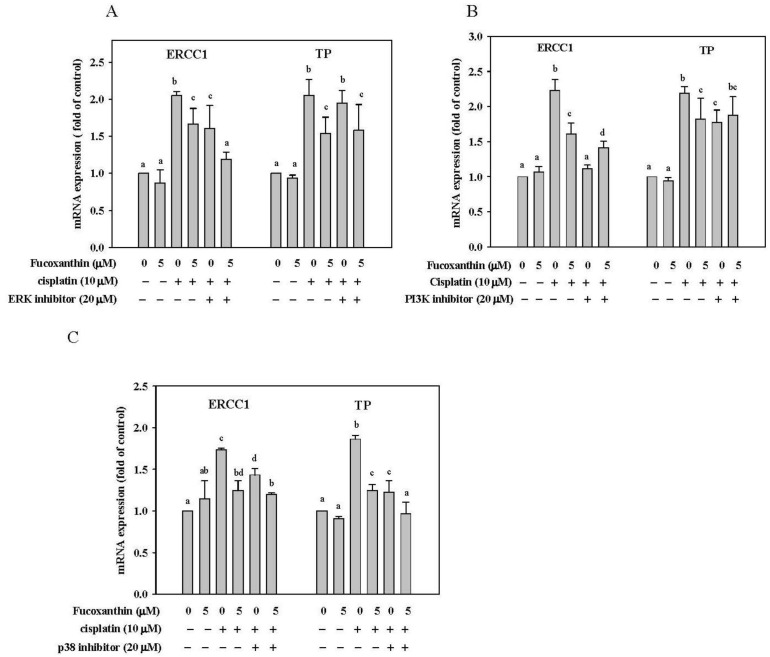
The level of ERCC1 and TP mRNA in HepG2 cells pretreated with fucoxanthin (5 μM) for 24 h followed by incubation with a specific inhibitor (20 μM) for 1 h and then treated cisplatin (10 μM) for 24 h. (**A**) ERK inhibitor (PD98059); (**B**) PI3K inhibitor (LY294002); (C) p38 inhibitor (SB203580). Values are means ± SD, *n* = 3; means without a common letter differ significantly, *P* < 0.05.

We then determined whether the attenuation of fucoxanthin on ERCC1 and TP mRNA expression induced by cisplatin occur primarily through the inhibition of ERK, p38 and PI3K/AKT pathway. HepG2 cells were pre-incubated with fucoxanthin (5 μM) for 24 h followed by incubation with ERK inhibitor (PD98059; 20 μM), p38 inhibitor (SB203580; 20 μM) or PI3K inhibitor (LY294002; 20 μM) for 1 h and then with cisplatin (10 μM) for 24 h. The concentration of 5 μM fucoxanthin was chosen because it only produced a slight inhibition of mRNA expression for ERCC1 and TP (see Figure 4). We found that the combination of fucoxanthin, cisplatin and the ERK inhibitor or PI3K inhibitor synergistically inhibited ERCC1 mRNA expression but not TP mRNA expression (Figure 6A,B). In contrast, the combination of fucoxanthin with p38 inhibitor enhanced the inhibition of mRNA expression of TP but not ERCC1 (Figure 6C). Thus, the results reveal that fucoxanthin may inhibit ERCC1 mRNA expression through ERK and PI3K/AKT pathway but may inhibit TP mRNA expression through p38 pathway. 

## 3. Discussion

The main question addressed by this study was whether a combination of fucoxanthin and cisplatin improves the efficacy of chemotherapy and enhances the inhibition of cell proliferation. Our data demonstrate for the first time that fucoxanthin in combination with cisplatin inhibits the cell proliferation of human hepatoma HepG2 cells and that this combined effect involves NFκB mediated-pathways and attenuation of the DNA repair gene expression induced by cisplatin. 

A probable mechanism by which fucoxanthin improves the efficacy of cisplatin chemotherapy is through the inhibition of NFκB expression and the increase in NFκB-regulated Bax/Bcl-2 mRNA expression. NFκB is known to suppress apoptosis through the loss of pro-apoptotic factors (e.g., functional p53 or Bax) or the activation of anti-apoptotic factors such as Bcl-2, Bcl-XL or IAPs (Inhibitors of Apoptosis Proteins) to block caspase-8 activation [24]. Moreover, NFκB binding sites were found in the promoter of Bcl-2, Bcl-xL, and survivin [49], suggesting that these apoptotic factors may be regulated by NFκB. Overexpression of the antiapoptotic molecules Bcl-2 or Bcl-xL can cause resistance to anticancer drugs [50]. We found that fucoxanthin pretreatment attenuated cisplatin-induced DNA-binding activity of NFκB, restored cisplatin-inhibited IκB-α***-***phosphorylation, and increased the ratio of Bax/Bcl-2 mRNA expression, rendering cancer cells sensitive to apoptosis induced by cisplatin. Many NFκB inhibitors have been identified *in vitro *such as proteasome inhibitors, genistein, parthenolide, flavopiridol and gliotoxin, enhance the cytotoxic effect of anticancer agents [29]. In the present study, we found that fucoxanthin strongly attenuated cisplatin-induced NFκB activation, suggesting that fucoxanthin may be an NFκB inhibitor. 

Another probable mechanism by which fucoxanthin improves the efficacy of cisplatin chemotherapy is through the inhibition of mRNA expression of DNA repair genes ERCC1 and TP. Tumor DNA repair capacity is frequently increased as an inherent cellular mechanism for evading cell death as a result of administration of chemotherapeutic drugs. Increased expression of ERCC1 in several cancers has been associated with more efficient removal of DNA adducts induced by platinum, leading to clinical resistance to cisplatin-based chemotherapy [51]. Depletion of endogenous ERCC1 expression by si-ERCC1 RNA transfection was shown to significantly enhance the cytotoxicity of cisplatin [52]. In the present study, we found that fucoxanthin pretreatment significantly attenuated cisplatin-induced ERCC1 and TP mRNA expression. Recently, Tsai *et al.* [53] have shown that curcumin downregulates the expression levels of TP and ERCC1, which helps overcome platinum resistance in cancer cells. 

MAPK family and PI3K/AKT pathways play important roles in the regulation of cell proliferation, differentiation, apoptosis and DNA repair pathways [54]. Protein kinase C and Ras have been suggested to be involved in the activation of ERK1/2 by cisplatin [55]. AKT may promote cell survival by phosphorylating and inactivating the pro-apoptotic proteins BAD (Bcl-2-associated death protein) and caspase-9 [56]. It has been reported that the inactivation of NFκB-binding activity sensitizes human ovarian cancer cells to cisplatin *in vitro * [57] and that the combined therapy of cisplatin with a PI3K inhibitor enhances the apoptotic effect of cisplatin *in vivo * [58]. In this study, we found that cisplatin activated the phosphorylation of ERK1/2, p38, AKT and PI3K in human hepatoma HepG2 cells and that fucoxanthin pretreatment attenuated cisplatin-induced phosphorylation of all these signaling molecules to sensitize cancer cells to apoptosis induced by cisplatin. With regard to the DNA repair system, it has been shown that specific MERK/ERK and PI3K inhibitors prevent ERCC1 induction, whereas JNK and p38 inhibitors are without effects in human hepatoma cells [59]. Interestingly, curcumin and emodin, which are natural anthraquinone derivatives found in the roots and rhizomes of numerous plants, were found to enhance cisplatin-induced cytotoxicity via downregulation of ERCC1 and inactivation of ERK1/2 in non-small cell lung cancer [52]. The inhibition of the PI3K pathway on ERCC1 basal expression has been confirmed by using shRNA against FRAP/mTOR, a key kinase involved in nucleotide excision repair pathway [60]. TP, a key enzyme in the pyrimidine nucleoside salvage pathway, was also found to be regulated by NFκB- and p38 MAPK-mediated signaling in patients with nasopharyngeal carcinoma [61]. In the present study, we found that fucoxanthin inhibited ERCC1 mRNA expression through the ERK and PI3K/AKT pathways, whereas this carotenoid inhibited TP expression through the p38 pathway, as evidenced by using specific inhibitors. The results indicate that the improved chemotherapeutic efficacy of cisplatin by fucoxanthin may also involve inhibition of mRNA expression of some DNA repair genes through downregulation of ERK, p38, and PI3K/AKT pathways. 

## 4. Experimental Section

### 4.1. Materials

Dulbecco’s modified eagle medium (DMEM), fetal bovine serum (FBS), trypsin, penicillin, sodium pyruvate, and non-essential amino acids (NEAA) were purchased from GIBCO/BRL (Maryland, MD, USA). MAPK/extracellular signal-regulated kinase (ERK) 1/2, c-Jun NH2-terminal kinase (JNK)/stress-activated protein kinase and p38 MAPK proteins and phosphorylated proteins, phosphatidylinositol 3-kinase (PI3K)/AKT, ERK inhibitor (PD98059), p38 inhibitor (SB203580) and PI3K inhibitor (LY294002) were purchased from Cell Signaling Technology (Beverly, MA). NFκB activation inhibitor was purchased from Merck Millipore (Billerica, MA, USA). Fucoxanthin was extracted from *Undaria pinnatifida* and purified, as we reported previously [62]. The purified fucoxanthin was dissolved in ethanol to a final concentration of 10 mM as the stock solution. Before the experiment, fucoxanthin solutions were prepared freshly in a mixture of ethanol and FBS (1:9), as adopted from the preparation of lycopene solution [63]. 

### 4.2. Cell Cultures

The human hepatoblastoma HepG2 cell line was obtained from Food Industry Research and Development Institute (FIRDI, Hsinchu, Taiwan) and maintained in DMEM supplemented with 10% fetal bovine serum without antibiotics under 5% CO_2_ at 37 °C.

### 4.3. Assessment of Cell Viability

Cell viability was evaluated using the modified acid-phosphatase (ACP) assay, with *p*-nitrophenyl phosphate (PNPP) disodium salt as a substrate. The cell culture media were aspirated, and the cells were washed with phosphate-buffered saline (PBS). Following the wash, 100 μL of the ACP reagent (0.1 M sodium acetate (pH 5.5), 0.1% Triton X-100, and 10 mM PNPP) was added. After 1 h of incubation at 37 °C, the enzyme activity was stopped by adding 10 μL of 1 N NaOH, and the enzyme activity was determined photometrically at a wavelength of 405 nm [64].

### 4.4. Real-Time Polymerase Chain Reaction

Total RNA in cell cultures was extracted with REzol reagent (PROtech Technologies, Inc., Placentia, CA, USA), and 1 μg of total RNA was reverse-transcribed by using oligo-dT as a primer in 20 μL reverse-transcription solutions containing 1 μL reverse transcriptase (Promega, Sunnyvale, CA, USA). Real-time PCR performed with a Corbett instrument (Applied Biosystems, Carlsbad, CA, USA) using SYBR Green Master Mix (ProTech, Placentia, CA, USA) according to the manufacturer’s instructions. In all real-time PCR experiments, both a non-template control (NTC) and a standard curve were amplified, as well. The RNA abundance was normalized to β-actin RNA in each sample. The primers used in this study were as follows: ERCC1 forward 5′-CCCTGGGAATTTGGCGACGTAA-3′, reverse 5′-CTCCAGGTACCGCCCAGCTTCC-3′; TP forward 5′-AGCTGGAGTCTATTCCTGGATT-3′, reverse 5′-GGCTGCATATAGGATTCCGTC-3′; β-actin forward 5′-GTGGGGCGCCCCAGGCACCA-3′, reverse 3′-CACCCCGCGGGGTCCGTGGT-5′.

### 4.5. Western Blotting

Protein expression of MAPK family (ERK, p38, JNK, p-JNK, p-ERK and p-p38) and PI3K/AKT (PI3K, AKT and p-AKT) was measured by Western blotting. In cell culture experiments, the medium was removed and cells were rinsed with PBS twice. After the addition of 0.5 mL of cold RIPA buffer and protease inhibitors, cells were scraped and followed by a mild vortexing at 0 °C for 20 min. The cell lysates were then subjected to a centrifugation of 10,000 rpm for 30 min at 4 °C. Total protein (50 μg) from the supernatant was resolved on SDS-PAGE and transferred onto a PVDF membrane. After blocking with TBS buffer (20 mmol/L Tris-HCl, 150 mmol/L NaCl, pH 7.4) containing 5% nonfat milk, the membrane was incubated with monoclonal antibody followed by incubation with horseradish peroxidase-conjugated anti-goat IgG, and then visualized using an ECL chemiluminescent detection kit (Amersham, Sweden). The relative density of the immunoreactive bands was quantitated by densitometry (Gel Pro Analyzer TM, version 3.0, Media Cybernetics, Rockville, MD, USA).

### 4.6. Preparation of Nuclear Extracts and Electrophoretic Mobility Shift Assay (EMSA)

Nuclear protein extracts (5 μg) were prepared according to the modified method of a previous study [65]. Binding activities of transcription factors including NFκB were analyzed by EMSA. Electrophoretic mobility shift assay (EMSA) was performed with LightShift Chemiluminescent EMSA Kit (Pierce Biotechnology, Rockford, IL, Country), as described previously [66]. The NFκB consensus oligonucleotide probe (5′-AGTTGAGGGGACTTTCCCAGGC-3′) was end-labeled with biotin (Sangon, Shanghai, China). Briefly, nuclear extract (5 μg) was incubated with 10 ng NFκB (p65) probe. For the cold probe assay, 40 ng of unlabeled (cold) NFκB probe was mixed with sample 5 min before adding 10 ng biotin-labeled NFκB probe. Protein-DNA complexes were then resolved by non-denaturing polyacrylamide gel electrophoresis (PAGE). After blocking, avidin-HRP was applied and detected by enhanced chemiluminescence (ECL, Amersham). The relative NFκB levels were quantitated by Matrox Inspector 2.1 software.

### 4.7. Transfection and Luciferase Reporter Gene Assays

HepG2 cells (1.8 × 10^4^ cells/well) were plated in 96-microwell-white-plates (Nalge Nunc, Rochester, New York, NY, USA) before transfection. The NFκB plasmid vector (pGL4.32 (luc2P/NF-κB-RE/Hygro)) contains five copies of an NFκB response element was purchased from Promega (Sunnyvale, CA, USA). Transfection of NFκB plasmid vector (0.15 μg) into HepG2 cells was performed using TransIL-LT1 Transfection Reagent (Mirus, Madison, WI, USA), and in all experiments, the pRL-TK Renilla reporter vector (0.02 μg) (Promega, Sunnyvale, CA, USA) was used as an internal control. The cells were then incubated with cisplatin (10 μM) for 16 h after pretreatment with fucoxanthin (1–10 μM) for 24 h. Renilla and firefly luciferase activities were measured using the Dual-Luciferase Reporter Assay System (Promega, Sunnyvale, CA, USA).

### 4.8. Statistical Analysis

All experiments were repeated at least thrice. Values are expressed as means ± SD and analyzed using one way ANOVA followed by LSD test for comparisons of group means, when the F ratios were significant. All statistical analyses were performed using SPSS for Windows, version 10 (SPSS, Inc., Armonk, NY, USA); a *P* value < 0.05 is considered statistically significant.

## 5. Conclusions

In conclusion, the present study demonstrates that pretreatment with fucoxanthin improves the chemotherapeutic efficacy of cisplatin by enhancing the inhibition of cell proliferation of human hepatoma HepG2. These effects of fucoxanthin may involve the inhibition of NFκB expression and the increase in Bax/Bcl-2 mRNA ratios regulated by NFκB, as well as the decrease of DNA repair systems regulated by ERK, p38 and PI3K/AKT, leading to sensitized cancer cells to apoptosis induced by cisplatin. The results suggest that the combined treatment of fucoxanthin and cisplatin may provide a novel therapeutic approach to decrease cisplatin-induced drug resistance.

## Supplementary Files

Supplementary File 1 Supplementary Information (PDF, 42 KB)
