# Supplementary material for: Fucoxanthin Enhances Cisplatin-Induced Cytotoxicity via NFκB-Mediated Pathway and Downregulates DNA Repair Gene Expression in Human Hepatoma HepG2 Cells
Source: Mar Drugs. 2013 Jan 8;11(1):50–66. doi: 10.3390/md11010050 (PMC3564157; doi:10.3390/md11010050)

Supplementary Information

Figure S1. Cell proliferation of HepG2 cells incubated with fucoxanthin (1–10 μM) for 24 h and 48 h. Values are means ± SD, $n = 3$; means without a common letter differ significantly, $P < 0.05$.

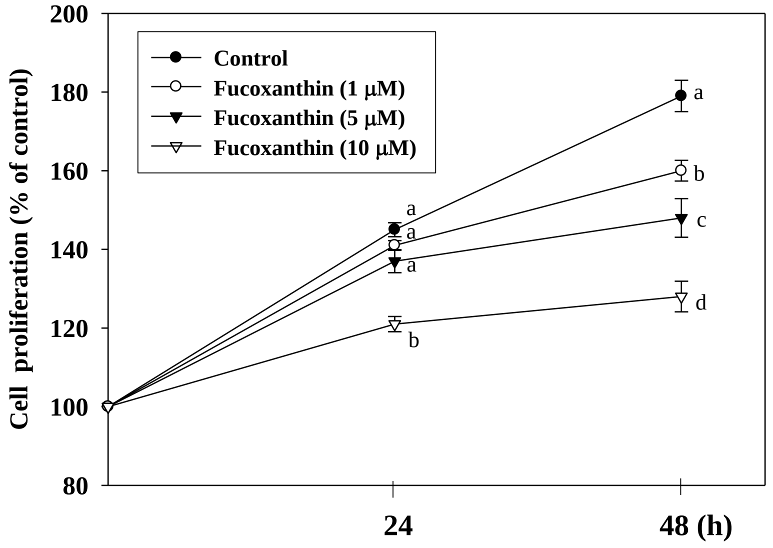

Supplement: Supplementary File 1 — Supplementary Information (PDF, 42 KB) [file marinedrugs-11-00050-s001.pdf]
